# Blood flow restriction with different loads versus moderate intensity resistive exercises in patients with knee osteoarthritis: a single-blind randomized controlled trial

**DOI:** 10.1186/s13102-026-02068-y

**Published:** 2026-10-07

**Authors:** Afaf Mohamed Tahoon, Farah Fouad Gomaa El Gamal, Shaimaa Mohamed Metawee, Noha Khaled, Nasr Awad Abdelkader

**Affiliations:** 1 Department of Physical Therapy for Musculoskeletal Disorders and its Surgery, Faculty of Physical Therapy, Cairo University, Giza, Egypt; 2 Department of Physical Therapy for Musculoskeletal Disorders and its Surgery, Faculty of Physical Therapy, Cairo University, Giza, Egypt; 3 Department of Physical Therapy, Al-Gomhoria General Hospital, Ministry of Health and Population, Alexandria, Egypt; 4 Department of Basic Sciences, Faculty of Physical Therapy, Sinai University, Ismailia, Egypt; 5 Department of Biomechanics, Faculty of Physical Therapy, Cairo University, Giza, Egypt; 6 Department of Physical Therapy for Musculoskeletal Disorders and its Surgery, Faculty of Physical Therapy, Cairo University, Giza, Egypt

**Keywords:** Blood flow restriction training, Blood flow restriction exercise, BFR therapy, Knee osteoarthritis, Resistance training, Rehabilitation exercise, Limb occlusion pressure

## Abstract

**Background:**

Knee osteoarthritis (KOA) is a common condition causing pain, muscle weakness, and functional impairment. In addition, many patients cannot tolerate resistance training due to pain. Blood flow restriction (BFR) has been proposed as a low-load alternative strategy to improve muscle function, but the effectiveness of passive BFR application remains unclear in KOA. Therefore, this study compared passive BFR at 50% and 70% limb occlusion pressure (LOP) with moderate-intensity resistance training (MI-RT) in KOA.

**Methods:**

A three-arm, parallel groups, single-blind randomized controlled trial was conducted on 75 Patients, aged 45–60 years, diagnosed with KOA. They were randomly assigned to receive 16 sessions (twice weekly for eight weeks) for either the study groups (BFR-50% LOP; BFR-70% LOP) or the control group (MI-RT). The primary outcomes were quadriceps torque and normalized torque. Pain intensity, functional mobility, functional ability, KOA impact, Rate of Perceived Exertion, and global perceived improvement were secondary outcomes. Measures were recorded at baseline, 4, and 8 weeks.

**Results:**

All interventions produced significant improvements over time across all outcomes (*p* < 0.001). Compared with both BFR groups, MI-RT produced greater increases in quadriceps torque than BFR-50% LOP (MD = 18.57 N.m, *p* < 0.001) and BFR-70% LOP (MD = 13.81 N.m, *p* = 0.004) at week 8, with significant increases in perceived exertion, whereas no changes in exertion were observed in either BFR group. The BFR-70% LOP group demonstrated greater improvement in lower-extremity functional ability than MI-RT (MD = 6.8 points, *p* = 0.02) and BFR-50% LOP (MD = 15.9 points, *p* < 0.001). However, no significant between-group differences were observed for pain intensity, KOA impact, functional mobility, or perceived improvement.

**Conclusion:**

MI-RT was the most effective intervention for improving quadriceps strength, whereas passive BFR at 70% LOP yielded the greatest improvements in lower-extremity functional ability while requiring less perceived exertion. These findings suggest that passive BFR at 70% LOP may be a feasible rehabilitation option for patients with knee osteoarthritis who cannot tolerate resistive exercise. Passive BFR at 50% LOP produced smaller functional improvements than the other interventions.

**Trial registration:**

The study was registered at ClinicalTrials.gov (No: NCT06637124) on September 24, 2024. URL:https://share.google/fuSDVPVt2pxG7M1HU.

## Introduction

Knee osteoarthritis (KOA) is a highly frequent chronic musculoskeletal disorder globally and a significant contributor to disability in older adults [1]. The Global Burden of Disease estimates that osteoarthritis impacted around 595 million individuals in 2020, with the knee accounting for nearly four-fifths of the global osteoarthritis burden [2]. KOA is marked by increasing deterioration of articular cartilage and alterations in subchondral bone in addition to inflammation of the synovial membrane, leading to pain, stiffness, difficulties in performing daily activities, and reduced functional capacity [3]. Quadriceps muscular weakness and reduced lower-limb function are the major KOA impairments that aggravate symptoms and disease progression [4]. 

Non-pharmacological management, especially physical therapy, is fundamental to KOA treatment in the clinical practice guidelines [5, 6]. Therapeutic exercises are considered the cornerstone of KOA physical therapy management [7]. The International Osteoarthritis Research Society recommends strengthening training as a major component of KOA physical therapy management that may slow disease progression [5]. Consistent with these recommendations, randomized clinical trials have shown that different strengthening exercise approaches, including open- and closed-kinetic chain exercises, improve pain, muscle strength, and physical function in individuals with KOA [8]. The American College of Sports Medicine recommends moderate-intensity resistive training (MI-RT) at 60 to 70% of one-repetition maximum (1RM) to optimize muscle strength and hypertrophy [9]. This intensity promotes myofibrillar protein synthesis, supporting muscle mass and mitigating age-related loss [10]; however, many KOA patients struggle with adherence to the traditional MI-RT programs due to pain, obesity, joint instability, and prior injury, which reduce tolerance to mechanical loading and limit participation in moderate-to-high-intensity programs [11]. Additionally, excessive joint loading may accelerate degenerative changes, raising concerns about the feasibility of these interventions [12]. Consequently, alternative rehabilitation strategies that reduce mechanical joint loading while improving pain and physical function, including aquatic therapy, virtual reality-based rehabilitation, and blood flow restriction (BFR), have received increasing attention in the management of knee osteoarthritis [13, 14]. 

Among these approaches, BFR has demonstrated considerable potential as an adjunct in musculoskeletal rehabilitation through the application of an external cuff placed proximally on the limb to partially restrict arterial blood flow while substantially restricting venous return during rest or exercise [14]. Several systematic reviews [14–17] established beneficial effects of BFR on muscle strength, mass, pain, and function; however, findings remain inconsistent due to heterogeneity in protocols, particularly cuff pressure, exercise intensity, and occlusion duration. Although the percentage of limb occlusion pressure (LOP) is considered a key determinant of the physiological stimulus and safety of BFR interventions, optimal pressure remains unclear, with evidence suggesting 40–50% LOP may balance efficacy and tolerability [18]. While higher ranges (50–80% LOP) may be required to achieve adaptations comparable to high-intensity resistive training (HI-RT) [19]. However, comparative evidence evaluating different occlusion pressures in patients with knee osteoarthritis remains limited. Moreover, most studies have focused on BFR combined with exercise, whereas passive BFR remains underexplored despite evidence suggesting that it can attenuate muscle atrophy and strength loss in immobilized individuals [20, 21]. 

Passive BFR may be particularly relevant for individuals with KOA who are unable to tolerate conventional resistance exercise because of pain, joint irritability, or reduced physical capacity. By inducing local hypoxia and metabolic stress without external mechanical loading, passive BFR may stimulate physiological adaptations through cellular swelling, anabolic signaling, and reperfusion-mediated responses while minimizing stress on the affected joint, making it a potentially valuable rehabilitation option when active exercise is not feasible [22, 23]. 

This highlights a critical gap in understanding its potential role in improving clinical and muscular outcomes in individuals with KOA who are unable to tolerate active exercise.

To the best of our knowledge, no published study has directly compared passive BFR applied at different occlusion pressures with MI-RT in patients with knee osteoarthritis. Therefore, this study aimed to evaluate whether passive BFR could serve as an effective alternative to conventional MI-RT and to compare the clinical effects of two individualized occlusion pressures (50% and 70% LOP) in patients with KOA. We hypothesized that passive BFR performed at 70% LOP would produce greater improvements in quadriceps strength and functional outcomes than passive BFR performed at 50% LOP.

## Methods

### Study design, setting, and participants

A prospective, parallel, single-blind randomized controlled study with an equal allocation ratio of 1:1:1 was conducted in well-equipped outpatient rehabilitation hospitals in Alexandria, Egypt, within dedicated physical therapy departments designed to support both conventional and advanced rehabilitation interventions. The intervention and assessments were carried out by a single licensed orthopedic physical therapist with more than 10 years of clinical experience and prior training and practical expertise in BFR application, ensuring consistency in treatment delivery and minimizing inter-therapist variability. The physical therapy departments were equipped with standard rehabilitation infrastructure, including adjustable treatment plinths, patient trolleys, and private treatment areas to ensure patient comfort, confidentiality, and safety during assessment and intervention procedures. This study was reported in compliance with the Consolidated Standards of Reporting Trials statement. The study adheres to CONSORT guidelines, and a completed CONSORT checklist is submitted as an additional file.

Ethical approval was obtained from the Research Ethics Committee of the Faculty of Physical Therapy, Cairo University (Approval No. P.T.REC/012/005029). The trial was subsequently registered at ClinicalTrials.gov (Identifier: NCT06637124) on September 24, 2024. The study was performed in accordance with the guidelines of the Declaration of Helsinki. All participants received standardized verbal instructions outlining the intervention procedures specific to their assigned group. A written consent form was signed by all patients before their enrollment in this study, highlighting their right to withdraw or decline participation at any time, in addition to the confidentiality of any participant’s information or images obtained, which was strictly maintained, with all identifying information securely stored and accessible only to authorized research personnel.

Inclusion criteria for this study included patients aged 45–60 years [24] with a primary KOA diagnosis according to the American College of Rheumatology radiological and clinical classification [25], having symptomatic KOA of ≥ 3 months, reported moderate to severe knee pain quantified on the Visual Analog Scale (VAS) as 4/10 or above, moderate disability on Arabic version of Western Ontario and McMaster Universities Osteoarthritis Index (WOMAC) with total score of 36–60, and moderate physical activity levels between 3.0 and 5.9 on the International Physical Activity Questionnaire [26]. Knee osteoarthritis severity was classified using the Kellgren–Lawrence system on weight-bearing anteroposterior radiographs. The principal researcher assigned the Kellgren–Lawrence grade using standardized criteria [27] with confirmation from the referral report when available. Only patients with grades II–III were enrolled.

Exclusion criteria included any previous knee surgeries or fractures of the lower limb, knee arthroscopy, or intra-articular injection within the previous 6 months before enrollment in the study [28], psychological, cognitive, or neurological disorders that may affect a subject’s performance in the study, congenital lower limb deformity, or having participated in a rehabilitation program in the past 6 months [29], secondary osteoarthritis, rheumatoid arthritis history, or even any other chronic inflammatory joint disease with laboratory evidence supporting such a diagnosis, knees with clinically significant synovitis (warmth, swelling, or redness) suggesting an inflammatory process beyond typical KOA, history, and/or physical examination findings compatible with knee internal derangement (ligaments or meniscal injuries) [30]. Additional exclusion criteria included any contraindications to BFR, such as pregnancy, cardiovascular disorders, current use of a medication that would affect blood pressure response, previous vascular surgery or vascular disease, hypertension, hemophilia or other bleeding/clotting disorder, venous thromboembolism, cerebral stroke or infarction, diabetes, and peripheral neuropathy [31]. 

### Sampling, randomization, allocation concealment, and blinding

Patients were recruited as a convenience sample via advertisements in the outpatient rehabilitation hospitals, direct patient referrals from orthopedic surgeons, and social media posts on Instagram and Facebook. Participants were assigned to one of three study groups (MI-RT, BFR 50% LOP, BFR 70% LOP) using simple randomization. The random allocation sequence was generated using Microsoft Excel by an independent researcher who was not involved in participant recruitment or trial implementation. Allocation concealment was ensured using sequentially numbered, opaque, sealed envelopes prepared by an independent researcher.

The investigator responsible for participant enrollment had no access to the allocation sequence. Participants were assigned to separate treatment schedules and rooms to minimize cross-group communication; however, participant blinding was not feasible due to the nature of the interventions. Both the outcome assessor and the statistician were blinded to group allocation.

### Outcome measures

The quadriceps torque and normalized torque were the primary outcome measures. Quadriceps torque was measured using a portable handheld dynamometer (Lafayette Handheld Dynamometer Model 01165 A, USA), a device demonstrated to provide reliable and valid measurements of lower-limb isometric muscle strength [32]. The participants sat on the table with their hips and knees flexed approximately at 90°, and a non-elastic strap was used to secure the dynamometer 5 centimeters proximally to the ankle joint. They were asked to perform 3 maximum voluntary isometric contractions of the quadriceps; each contraction lasted about 5 s, with a 30-second rest period between contractions. The average of the three attempts was recorded, and the torque was calculated by multiplying the observed force by the length of the lever arm. The torque measurements were normalized in relation to body weight to facilitate inter-subject comparison, thereby offering a standardized method to measure muscle strength [33, 34]. 

The secondary outcome measures were pain intensity, functional mobility, functional ability, KOA impact, Rate of perceived exertion (RPE), and global perceived improvement. The Visual Analogue Scale (VAS) is a reliable, valid, sensitive tool for quantifying pain intensity. The VAS consists of a 10 cm horizontal line, with the two ends representing the extreme limits of pain perception, ranging from “no pain” to “worst possible pain.” The VAS has demonstrated excellent reliability, with an intraclass correlation coefficient of 0.93 [35]. 

Functional mobility was measured using the Timed Up and Go (TUG) test, which is a reliable, valid, time-efficient, and safe method for assessing functional mobility [36]. It was used to measure and record the duration required for the patients to stand up from a standard armless chair, walk a distance of three meters, turn around, return to the chair, and sit down again. TUG score was recorded in seconds, representing the total time taken from the moment of standing up until sitting down [36]. The Lower Extremity Functional Scale (LEFS) in Arabic form was used to assess functional ability. LEFS was a valid and reliable self-reported scale for patients who have musculoskeletal disorders of the lower limbs [37]. It is responsive to clinically meaningful changes over time, allowing detection of functional improvement or deterioration, and is therefore suitable for baseline assessment, rehabilitation monitoring, and outcome evaluation. The instrument comprises 20 items scored on a 5-point Likert scale (0–4), where 0 denotes severe difficulty or inability to perform the task and 4 indicates no difficulty. Total scores range from 0 to 80, with higher scores reflecting better functional status. Patients were instructed to self-report their capacity to perform activities such as walking, stair climbing, and running [38]. 

KOA’s impact on patients was measured using the valid and reliable Arabic version of the WOMAC [39]. Patients were instructed to read each question carefully and choose the response that best described their experience using a 24-item Likert scale divided into three sections: pain (5 items), stiffness (2 items), and physical function (17 items). The options include “none,” “mild,” “moderate,” “severe,” or “extreme.” The total score is obtained by summing the item scores within each subscale and then normalizing the result to a maximum of 96. Higher scores indicate greater levels of pain, stiffness, and functional limitation [40]. 

The RPE was evaluated using the Arabic-translated version of the OMNI scale. This version has demonstrated strong concurrent validity, showing correlations between RPE and %1RM in the range of 0.95 to 0.96 [41]. This scale includes eleven items that range from 0 (“extremely easy”) to 10 (“extremely hard”). Patients were instructed to rate their RPE at three time points: before, during, and after the activity, considering the sensations of exertion experienced in both their lower limbs and their breathing. Participants provided the number that represented their current level of effort at each evaluation point on the scale. The recorded scores were subsequently summed and averaged by dividing the total by the number of assessments, yielding a mean RPE value that represents the overall perceived exertion across the activity [41]. Global perceived improvement in osteoarthritis symptoms was assessed using the valid and reliable Global Rating of Change (GROC) scale. This fifteen-point Likert scale ranges from − 7 (very much worse) to + 7 (very much better). Patients were instructed to consider their overall perception of change across multiple domains, including pain, quality of life, and disability [42]. 

All primary and secondary outcome measures were assessed at baseline, week 4, and week 8 post-intervention, except for GROC, which was evaluated only after the intervention. Additionally, participants were required to discontinue the use of anti-inflammatory medications for one week before enrollment in the study [43]. 

### Intervention

#### Control group (MI-RT)

Patients in the control group underwent MI-RT at 60% of one-repetition maximum, which includes 4 exercises consisting of leg press, seated knee extensions, calf flexion, and leg curl [44]. The leg press exercise was performed using a 45° leg press machine, with the participant seated in an upright position and both feet placed on the footplate. From an initial position of knee flexion, participants were instructed to extend the affected limb nearly to full knee extension, followed by a controlled return to the starting position. For the knee extension exercise, participants were seated on a knee extension machine with the thigh stabilized and the knee positioned within a 90° to 0° range of motion. They were instructed to extend the affected knee nearly to full extension and then slowly return to the initial position in a controlled manner. During the leg curl exercise, participants were positioned prone on the leg curl machine with the knees fully extended in the starting position. They were instructed to perform knee flexion by drawing the heels toward the buttocks through the available range of motion, followed by a slow and controlled return to knee extension at the starting position. The calf raise exercise was performed using a seated calf raise machine. Participants maintained an upright posture with the lumbar spine in a neutral alignment and feet supported on the platform. From a neutral ankle position, they elevated the heels against a load corresponding to 60% of 1RM and then lowered them to the starting position slowly in a controlled manner.

All the above-mentioned exercises in the MI-RT protocol consisted of 3 sets of 12 repetitions at 60% of 1RM, with a two-minute rest period between sets [44]. A total of sixteen treatment sessions (two sessions/week for eight weeks) in accordance with exercise guidelines for KOA [5]. In order to monitor changes in muscular strength over time, participants’ 1RM was reassessed at 4 and 8 weeks following the initial evaluation. Periodic reassessment allows adjustment of training loads in accordance with strength gains, ensuring maintenance of the prescribed relative intensity and providing adequate mechanical stimulus for continued adaptation [45]. As 1RM values increased, absolute loads were recalculated to preserve the target training intensity, thereby supporting progressive overload while minimizing excessive fatigue or injury risk [9, 46]. 

### Study groups (BFR-50% LOP; BFR-70% LOP)

The intervention groups received passive BFR at either 50% or 70% of LOP. BFR training consisted of 16 supervised sessions, conducted twice weekly over eight weeks [47]. The BFR was applied using the Smart Cuff® 4.0 (USA), a device classified as a Class I medical device by the United States’ Food and Drug Administration. The system allows automated, limb-specific determination of optimal occlusion pressure to ensure individualized and precise pressure application. A wearable BFR therapy device is a valid tool for assessing LOP; when compared with the gold standard Doppler ultrasound, the device showed good agreement, with an ICC of 0.85 and a mean difference of 4.1 ± 13 0.8 mmHg, indicating that its measurements are generally close to the Doppler standard. The device exhibited excellent reliability, with interrater and test-retest ICCs of 0.97 and 0.94, respectively [48]. A standardized cuff placement was placed at a location equivalent to 33% of the distance distal from the inguinal crease to the patellar superior edge [49]. LOP was estimated by the device’s automatic LOP calibration system (iBFR™), which calculates LOP via a proprietary algorithm within approximately 10 s. To account for potential changes in limb circumference, vascular characteristics, or systemic blood pressure, LOP assessment was repeated every two weeks throughout the intervention period. Based on the individualized LOP values, occlusion pressure was set according to the allocation group at either 50% or 70% of LOP. During each session, pressure was maintained for approximately 5 min across five cycles, separated by 3-minute reperfusion periods, while participants remained seated at rest without performing exercise [20]. 

All participants were closely monitored for any adverse events, such as musculoskeletal injury, any unexpected pain sensation, or even pain attributed to the BFR or MI-RT interventions, that resulted in cancellation of the sessions. All interventions were delivered by a single physical therapist to ensure consistency of treatment administration and minimize variability.

## Sample size estimation and Statistical analysis

An a priori sample size was estimated using G*POWER statistical software (version 3.1.9.2; Franz Faul, Universität Kiel, Germany) based on a repeated-measures ANOVA (between–within interaction model) with 3 groups and three measurement time points, an alpha level of 0.05, and 95% statistical power. Assuming a medium effect size (f = 0.25) based on prior pilot trial data, the group × time interaction on knee extensor torque was set as the primary outcome, with a correlation among repeated measures of 0.5 and a non-sphericity correction of 0.75. A total sample of 66 participants would be required. To account for a potential 20% attrition rate, an increase of up to 75 participants (25 participants in each group).

Mixed-design Multivariate Analysis of Variance to evaluate the main effects of group (BFR-50% LOP, BFR-70% LOP, MI-RT) and time (baseline, 4 weeks, 8 weeks), as well as the group × time interaction, on the primary and secondary outcomes. Demographic and clinical characteristics (Age, sex, weight, height, BMI, and adherence) are reported as means and standard deviations, whereas affected side and dominant side are presented as counts and percentages. Between-group differences in gender, dominant side, and affected side were calculated using a chi-square test.

Analysis of covariance was applied to compare post-treatment functional ability and torque outcomes between groups while adjusting for baseline values. Effect sizes for pairwise between-group comparisons were calculated using Cohen’s d, computed as the adjusted mean difference divided by the pooled standard deviation of the corresponding groups.

All statistical analyses were conducted using IBM SPSS Statistics (IBM Corp., Armonk, NY, USA), with the level of statistical significance set at *P* < 0.05.

## Results

**Fig. 1:**
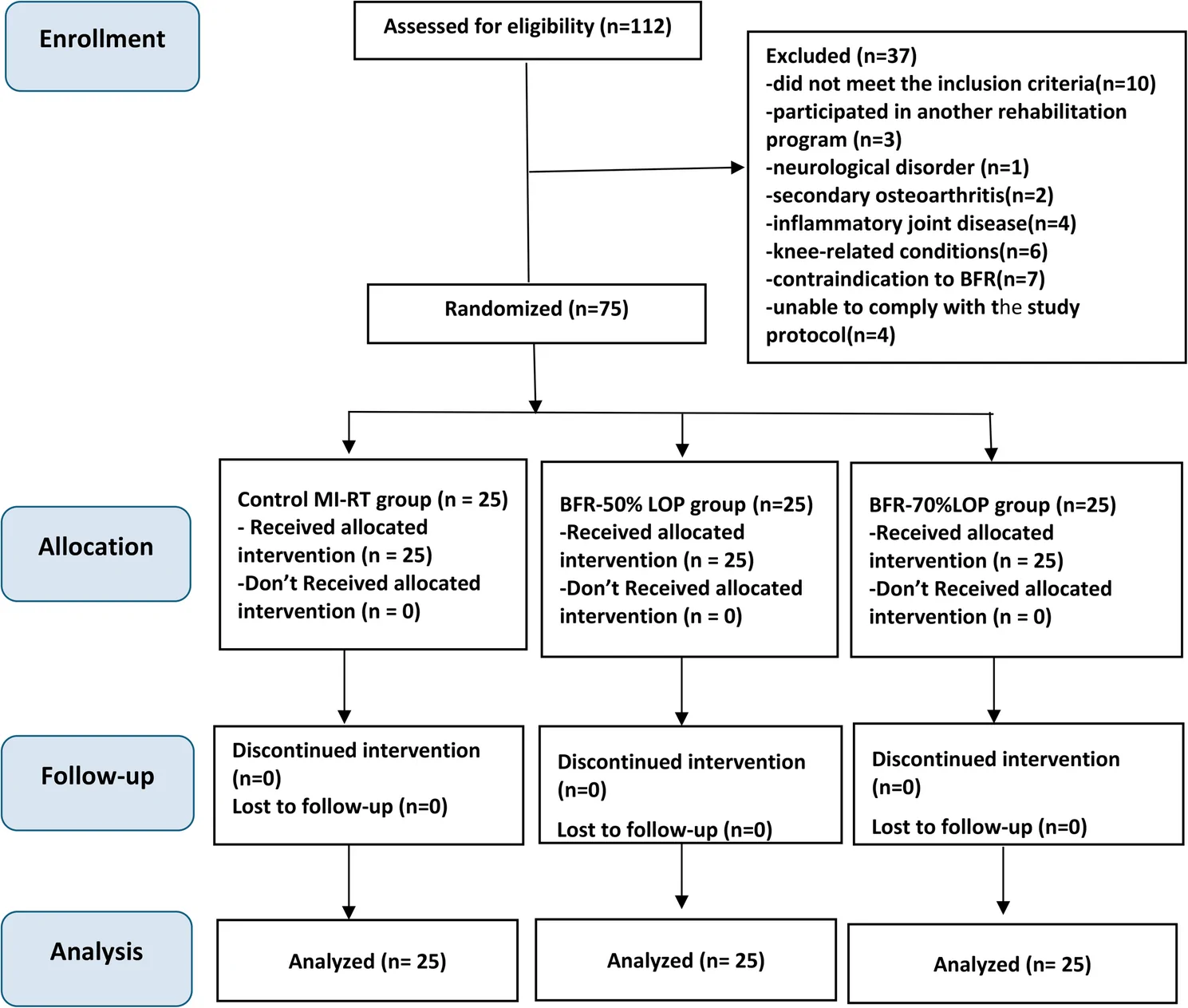
Flowchart for patients’ eligibility

Seventy-five out of 112 KOA patients enrolled in this study met the inclusion criteria; 37 participants were excluded for different reasons, as shown in the flowchart (Fig. 1).

All remaining participants received their assigned interventions and were analyzed in accordance with their original group allocation. As there were no missing outcome data or losses to follow-up, the analysis was effectively equivalent to an intention-to-treat analysis.

The demographic characteristics of all participants were compared across the intervention groups (Table 1). There were no statistically significant differences identified between the groups for any demographic variables except for gender, as the majority are female (66.6%). Comparison between male and female participants revealed statistically significant differences in functional ability, pain, and torque results (normalized and non-normalized) after adjusting post-treatment scores using ANCOVA with gender as a covariate. However, the adjusted mean differences did not surpass the established minimal clinically important difference, indicating that the observed between-group differences are unlikely to be of clinical relevance, regardless of the intervention type (mean differences = − 0.26, 7.6, 0.2, and 13.8, respectively). The overall multivariate analysis revealed a significant main effect of time (*p* < 0.001) and group (*p* < 0.001), as well as a significant group × time interaction (Wilks’ λ = 0.169, F (28, 118) = 6.02, *p* < 0.001, ηp² = 0.61). The results for outcomes are shown in Table 2.

**Table 1:** Comparison of demographic characteristics between the intervention groups

Item	MI-RT	BFR-50%LOP	BFR-70%LOP	Test value	*P*- value
	Mean ± SD	Mean ± SD	Mean ± SD		
Age (years)	50.56 ± 7.47	50.76 ± 7.61	49.92 ± 7.65	0.08	0.92
Weight (kg)	78.44 ± 13.45	86.16 ± 16.26	87.04 ± 13.40	2.69	0.08
Height (cm)	165.60 ± 7.71	162.48 ± 4.66	165.72 ± 7.75	1.79	0.17
BMI (kg/m²)	28.80 ± 5.84	32.63 ± 5.90	31.95 ± 6.19	2.92	0.06
Attendance	15.4 ± 0.82	15.84 ± 0.37	15.76 ± 0.52	5.4	0.07
Gender Male Female	13 (52%) 12 (48%)	3(12%) 22(88%)	9 (36%) 16 (64%)	9.1	0.01^*^
Affected side Right Left	16 (64%) 9 (36%)	13 (52%) 12 (48%)	13 (52%) 12 (48%)	0.97	0.61
Dominant side Right Left	21(84%) 4 (16%)	21(84%) 4(16%)	20 (80%) 5 (20%)	0.19	0.91

*SD* Standard deviation, *p-value* Probability value

* significant at *p* < 0.05

**Table 2:** Findings of primary and secondary outcomes before and after interventions among all groups

Outcome	Time point	MI-RT	BFR-50%LOP	BFR-70%LOP
Quadriceps torque (*N*.m)				
	Baseline	42.5 ± 12.75^a^	36.96 ± 7.32^a^	39.35 ± 10.99^a^
	Week 4	54.25 ± 15.3*^a^	42.96 ± 8.76*^b^	45.6 ± 11.54*^ab^
	Week 8	66.17 ± 19.78*^#a^	47.08 ± 8.55*^#b^	52.29 ± 12.63*^#b^
Quadriceps normalized torque (N.m/kg)				
	Baseline	0.57 ± 0.24^a^	0.43 ± 0.11^b^	0.47 ± 0.15^ab^
	Week 4	0.72 ± 0.29*^a^	0.50 ± 0.16*^b^	0.55 ± 0.17*^b^
	Week 8	0.88 ± 0.24*^#a^	0.55 ± 0.15*^#b^	0.62 ± 0.19*^#b^
Pain intensity (VAS)				
	Baseline	7.11 ± 0.78 a	6.92 ± 0.97a	6.78 ± 0.89a
	Week 4	6.11 ± 0.81*a	5.97 ± 1.03*a	5.84 ± 0.88*a
	Week 8	5 ± 0.89*#a	5.08 ± 0.96*#a	4.74 ± 0.94*#a
Functional mobility (TUG scores)				
	Baseline	10.26 ± 1.12^a^	10.21 ± 0.98^a^	10.39 ± 1.21^a^
	Week 4	8.94 ± 1.08*^a^	9.28 ± 1.26*^a^	9.39 ± 1.33*^a^
	Week 8	7.88 ± 0.85*^#a^	8.29 ± 1.2*^#a^	8.38 ± 1.49*^#a^
Functional ability (LEFS scores)				
	Baseline	29.04 ± 6.29^a^	25.4 ± 4.42^a^	30.8 ± 8.97^a^
	Week 4	41.68 ± 8.81*^a^	34.48 ± 4.46*^b^	46.32 ± 9.42*^a^
	Week 8	52.84 ± 10.93^*#a^	43.76 ± 5.18^*#b^	59.68 ± 10.17^*#c^
KOA impact (WOMAC scores)				
	Baseline	51.24 ± 7.11^a^	53.2 ± 4.85^a^	51.84 ± 8.72^a^
	Week 4	35.72 ± 6.65*^a^	40.16 ± 6.56*^a^	35.92 ± 9.83*^a^
	Week 8	21.24 ± 8.42^*#a^	28.84 ± 4.73^*#b^	19.76 ± 10.37^*#a^
Rate of perceived exertion (OMNI scale)				
	Baseline	4.76 ± 0.8	0 ± 0	0 ± 0
	Week 4	4.87 ± 0.61	0 ± 0	0 ± 0
	Week 8	5.07 ± 0.67^*^	0 ± 0	0 ± 0
Global perceived improvement (GROC scores)				
	Week 4	2.96 ± 0.68^a^	2.48 ± 0.59^b^	3.2 ± 0.82^a^
	Week 8	5.28 ± 1.06^*a^	4.44 ± 0.82^*b^	5.64 ± 0.86^*a^

*MI-RT* Moderate-intensity resistance training, *BFR* blood flow restriction, *LOP* limb occlusion pressure, *TUG* Timed up and go test, *LEFS* Lower extremity functional scale, *WOMAC* Western ontario and mcmaster universities osteoarthritis Index, *GROC* Global rating of change scale

Superscript letters (a, b, c) indicate significant between-group differences at the same assessment time (P < 0.05) based on Bonferroni-adjusted pairwise comparisons. Groups sharing the same superscript letter are not significantly different. A value labeled ab is not significantly different from either a value labeled a or b. Within-group comparisons are indicated by symbols, where * denotes a significant difference from baseline and # denotes a significant difference from the preceding time point (*P* < 0.05).

The primary outcomes were the quadriceps torque and normalized torque, their mean and standard deviation values at three time-points, which are listed in Table 2. At baseline, the study groups were similar in either quadriceps torque or normalized torque values, except between the BFR-50% LOP and MI-RT groups in normalized quadriceps torque (*p* < 0.05). Therefore, ANCOVA was performed for week 4 and week 8 comparisons, with pre-scores as a covariate.

Both quadriceps torque and normalized torque at weeks 4 and 8 are significantly higher in the MI-RT group than in both BFR groups. However, there is no statistically significant difference between the BFR groups. Quadriceps torque and normalized torque were significantly improved across all intervention groups as compared to baseline. Additionally, the highest torque and normalized torque values were observed in MI-RT, while the BFR-50% LOP group showed the lowest values.

The Secondary outcomes include Pain intensity, functional mobility, functional ability, KOA impact, rate of perceived exertion, and global perceived improvement. The pain intensity decreased significantly at the end of week 8 in all groups (*p* < 0.001), with the BFR 70% LOP group having the lowest value. However, the mean pain values were between the study groups at 3 time points (*p* > 0.05) (Table 2). Regarding self-reported OA symptom severity represented by the WOMAC score, all intervention groups demonstrated significant improvements from baseline, the BFR at 70% LOP group showed the greatest percentage change over the intervention period, at week-8, a significant difference between-group was observed, participants in the BFR-50%LOP exhibited significantly greater WOMAC scores compared with both the MI-RT group (MD = 7.6,

*p* = 0.004) and the BFR-70% LOP group (MD = 9.16, *p* < 0.001), demonstrating poorer self-reported physical function in the group of the BFR-50% LOP.

There was significant improvement across all groups in lower-limb functional ability, with the BFR-70% LOP group demonstrating the greatest change from baseline. There were no significant differences between groups at baseline, except between BFR groups, with a higher functional ability score in BFR-70% LOP (*p* < 0.05); therefore, ANCOVA was performed for week 4 and 8 comparisons. BFR-50% LOP had significantly lower LEFS scores at week-4 compared to MI-RT (MD = 11.8, *p* < 0.001) and BFR-70%LOP (MD = 7.2, *p* < 0.006). BFR-70% LOP had significantly higher LEFS scores at week-8 than MI-RT (MD = 6.8, *p* < 0.03), and 50% LOP (MD = 15.9, *p* < 0.001), while MI-RT had significantly higher scores than BFR 50% LOP (MD = 9.1, *p* < 0.002); Performance-based functional mobility, all groups showed a statistically significant improvement, while between group difference was not statistically significant. Perceived exertion was significantly increased in MI-RT at week-8 compared to baseline (*p* = 0.018), whereas the BFR groups were not included in the analysis because patients reported no exertion. All groups showed statistically significant differences in global perceived improvement, with BFR-50% LOP having significantly lower GROC scores at week 4 and week 8 compared to MI-RT (MD = 0.84, *p* < 0.006) and BFR-70% LOP (MD = 1.2, *p* < 0.001). No adverse events were reported in either group.

Clinical effects of each intervention protocol on all outcomes, as shown in Table 3, indicated by a significant group effect in ANOVA (except for pain intensity and functional mobility). Although perceived exertion also demonstrated a group effect, the scores for the BFR groups were zero; therefore, pairwise comparisons could not be conducted. MI-RT exhibited large effect sizes for quadriceps torque and normalized torque relative to the BFR groups. In KOA impact at week 4, the difference between MI-RT and BFR-50% LOP was not statistically significant; however, the effect size was clinically meaningful (0.67). At week 8, there was a large effect size for both MI-RT and BFR-70% LOP compared to BFR-50% LOP. Regarding functional ability, a large effect size was detected for either the 70%-BFR group or MI-RT compared to the 50%-BFR, while a moderate effect size was noted for comparison between BFR groups. A moderate-to-large effect size was observed in global perceived improvement for MI-RT and BFR 70% LOP relative to BFR 50% LOP.

**Table 3:** Pairwise comparison between groups and clinical effects of the interventions

Outcome	Time	Comparison	MD ± SE	*p* value	Effect size (d)
Quadriceps Torque (Nm)	**4 w**	A vs. B	10.84 ± 3.54	0.009*	0.87
		A vs. C	7.89 ± 3.54	0.087	0.58
		B vs. C	2.95 ± 3.54	1.00	0.29
	**8 w**	A vs. B	18.57 ± 4.13	0.001	1.22
		A vs. C	13.81 ± 4.13	0.004*	0.83
		B vs. C	4.76 ± 4.13	0.76	0.44
Normalized Torque	**4 w**	A vs. B	0.22 ± 0.06	0.001*	0.9
		A vs. C	0.18 ± 0.06	0.015*	0.78
		B vs. C	0.042 ± 0.06	1.00	0.26
	**8 w**	A vs. B	0.33 ± 0.07	0.001*	1.65
		A vs. C	0.26 ± 0.07	0.001*	1.20
		B vs. C	0.07 ± 0.07	0.95	0.42
KOA impact (WOMAC)	**4 w**	A vs. B	4.44 ± 2.2	0.15	0.67
		A vs. C	0.2 ± 2.2	1.00	0.02
		B vs. C	4.2 ± 2.2	0.18	0.51
	**8 w**	A vs. B	7.6 ± 2.3	0.004*	1.11
		A vs. C	1.56 ± 2.3	1.00	0.16
		B vs. C	9.16 ± 2.3	0.001*	1.13
Functional ability (LEFS score)	**4 w**	A vs. B	7.2 ± 2.2	0.006*	1.03
		A vs. C	4.6 ± 2.2	0.12	0.50
		B vs. C	11.8 ± 2.2	0.001*	1.50
	**8 w**	A vs. B	9.1 ± 2.6	0.002*	1.05
		A vs. C	6.8 ± 2.6	0.02*	0.64
		B vs. C	15.9 ± 2.6	0.001*	1.97
Perceived improvement (GROC score)	**4 w**	A vs. B	0.48 ± 0.18	0.01*	0.75
		A vs. C	0.24 ± 0.21	0.26	0.32
		B vs. C	0.72 ± 0.2	0.001*	1.01
	**8 w**	A vs. B	0.84 ± 0.26	0.006*	0.89
		A vs. C	0.36 ± 0.26	0.50	0.37
		B vs. C	1.20 ± 0.26	0.001*	1.43

*W* week, A: represents moderate intensity resistive training, B: BFR 50%LOP, C: BFR 70%LOP,

* MD: mean difference, *SE,* standard error, significant < 0.05.

## Discussion

The current study investigated the comparative efficacy of BFR at varying limb occlusion pressures (50% and 70% LOP) and MI-RT in patients with grade II–III KOA. The primary findings indicate that all three interventions yielded statistically significant improvements in quadriceps strength, pain intensity, and functional outcomes over eight weeks. These results underscore the clinical utility of both passive BFR and MI-RT in enhancing quadriceps torque and capacity for daily activity in this population.

While previous research has largely focused on BFR versus non-BFR interventions, fewer studies have directly examined whether varying BFR occlusion pressures influence pain and functional outcomes. Furthermore, the present study specifically examined the effects of BFR training as a standalone intervention, excluding the influence of concurrent exercise protocols.

A significant reduction in pain was observed across all groups, with no statistically significant differences between groups at any assessment time-point. This suggests that low-pressure passive BFR (50% LOP) may be as effective as higher pressures or traditional loading for pain management. The reductions in pain exceeded the commonly accepted minimal important change (MIC) of approximately 2 points [50] for the MI-RT and BFR 70% groups, indicating clinically meaningful pain relief. Although the BFR 50% group demonstrated a statistically significant reduction (1.84 points), this change was slightly below MIC threshold, suggesting that its clinical importance should be interpreted with caution.

These results align with previous literature [14, 51–52] which demonstrated the hypoalgesic effects of both BFR and moderate-to-high-load resistance exercise in patients with KOA. However, other evidence suggests a potential superiority of low-intensity resistance training (LI-RT) with BFR over conventional exercise in reducing pain [53, 54]. The differences between studies may be due to BFR application parameters, notably occlusion pressure, as pressures ranging from 40% to 80% of LOP with LI-RT may increase hypoalgesia via increasing metabolic stress and pain-modulating mechanisms.

While the outcomes are comparable, the underlying mechanisms likely diverge. MI-RT likely reduces pain by improving joint mechanics and patient self-efficacy. In contrast, BFR-induced hypoalgesia is hypothesized to stem from metabolic stress-induced hormonal responses, such as the release of growth hormone and beta-endorphins [55, 56]. as well as the activation of the endogenous opioid system and alterations in pain thresholds [57, 58]. Additionally, intermittent ischemia–reperfusion during BFR may contribute to pain modulation [58]. 

Regarding quadriceps torque and normalized torque, MI-RT produced significantly greater gains in quadriceps torque compared to passive BFR at both 50% and 70% LOP at the 4-week and 8-week marks. All groups improved, confirming that BFR is a viable strengthening approach for load-intolerant KOA patients. While BFR elevates metabolic stress, it may not entirely compensate for the absence of mechanical loading. Consequently, MI-RT may yield superior maximal strength improvements, particularly when joint tolerance permits. Interestingly, increasing occlusion pressure from 50% to 70% did not yield superior torque gains, suggesting that for strength alone, higher pressures may not provide an additive benefit over more comfortable, lower-pressure settings.

The greater improvements in normalized torque observed in the MI-RT group differ from current evidence suggesting that low-load resistance exercise combined with BFR may provide strength benefits comparable or superior to conventional resistance exercise in patients with KOA [53, 59, 60]. This discrepancy may be explained by the absence of a mechanical loading stimulus during passive BFR, together with differences in occlusion pressure prescription, training volume, intensity, and intervention protocols.

The Rating of Perceived Exertion increased significantly over time only in the MI-RT group. This elevation reflects the higher systemic and local demands associated with neural recruitment and metabolic stress in traditional resistance training [61, 62]. Conversely, the stability of RPE in the BFR groups highlights the primary advantage of this modality: achieving muscular adaptation with minimal external load and lower perceived effort, which is critical for clinical adherence in symptomatic KOA patients.

The current study demonstrated significant reductions in WOMAC total scores across all intervention groups, indicating progressive improvement in the overall impact of knee osteoarthritis from week 4 to week 8. Clinical relevance was interpreted using the reported minimal important difference of 7 points for the WOMAC Total score in patients with KOA [50]. The observed improvements exceeded this threshold, suggesting that they were not only statistically significant but also clinically meaningful. However, the greater reductions observed in the MI-RT and BFR 70% groups indicate superior clinical effectiveness compared with BFR at 50% occlusion pressure. These results align with existing literature [59, 60] that reported no significant difference between BFR along with LI-RT and conventional moderate or high intensity exercises. The 50% LOP group exhibited early functional gains but not long-term ones. By week 8, the 50% LOP group had significantly higher WOMAC ratings (indicating poorer function) than the MI-RT and 70% LOP groups. This shows that a lower occlusion stimulus may cause initial adaptations but be “under-dosed” for functional development. Thus, clinical care of KOA may require greater occlusion pressures of 70% LOP to optimize and sustain long-term functional results.

Significant longitudinal improvements in functional mobility, as assessed by the TUG test, were observed across all intervention groups. By week 8, all groups achieved TUG values below 9 s, indicating restoration of normal functional mobility and a reduced risk of falls [63]. In addition, the observed reductions in TUG time exceeded the proposed clinically important improvement of 0.8–1.4 s, originally established for patients with hip osteoarthritis and subsequently cited in reviews of osteoarthritis outcome measures, suggesting that the functional gains observed in the present study are likely to be clinically meaningful [6]. 

These findings align with established evidence [64] demonstrating that exercise interventions significantly improve mobility in patients with KOA. Historically, resistance training has been shown to reduce TUG times by improving neuromuscular coordination, lower-extremity strength, and patient confidence. Consistent with recent evidence [54, 59, 60], BFR-based exercise has been shown to have comparable improvements in functional mobility with conventional resistive exercises. However, a meta-analysis showed LI-RT with BFR produced significant improvements in walking ability when compared to LI-RT in middle-aged and older adults [65]. 

Significant improvements in the LEFS were observed across all groups, exceeding the established MIC by more than twofold [38], reinforcing the efficacy of both interventions in facilitating early functional gains through pain modulation, motor unit recruitment, and enhancement of movement confidence [6, 51]. Notably, at week 8, the passive BFR group at 70% LOP exhibited superior functional outcomes compared to those of the MI-RT and passive BFR 50% groups, suggesting that an adequately dosed BFR stimulus may surpass traditional loading in enhancing patient-reported functional ability when applied for a sufficient duration. In contrast, the 50% LOP group demonstrated significantly lower LEFS scores than the 70% LOP and MI-RT groups, likely due to reduced metabolic stress and fast-twitch fiber recruitment associated with lower pressures [49]. However, this group still achieved an improvement of 9.1 points, surpassing the MIC of 9 points and indicating functionally meaningful progress [38]. 

This finding partially contrasts with earlier evidence reporting comparable—but not superior—functional improvements between BFR and traditional resistance training [59, 60]. However, many of these studies used relatively low occlusion pressures, which may explain the absence of a clear advantage for BFR in earlier work. Conversely, the present findings partially agree with a network meta-analysis [53] which concluded that BFR combined with exercise produced greater improvements in knee function than exercise alone. The greater functional improvement observed with 70% LOP compared with 50% LOP suggests that a higher individualized occlusion pressure may generate a stronger physiological stimulus, potentially promoting greater neuromuscular adaptation and subsequent functional improvement.

The greater improvements in LEFS observed with passive BFR at 70% LOP compared with MI-RT may be explained by several physiological mechanisms. Unlike active BFR, passive BFR does not involve voluntary muscle contractions and is therefore unlikely to elicit the degree of motor-unit recruitment or muscle hypertrophy typically achieved with resistance exercise. Instead, repeated cycles of ischemia–reperfusion may induce metabolic stress through metabolite accumulation, cellular swelling, and attenuation of disuse-related muscle loss [19, 21, 66]. Passive BFR may also promote neuromuscular adaptations by modulating group III and IV afferent feedback, thereby attenuating arthrogenic muscle inhibition and facilitating voluntary muscle activation during functional activities [67]. Furthermore, cuff deflation induces reactive hyperemia and increased vascular shear stress, which may improve endothelial function, nitric oxide bioavailability, and local microvascular perfusion, potentially enhancing oxygen and nutrient delivery to the affected tissues [68, 69]. Collectively, these metabolic, neuromuscular, and vascular responses may contribute to improved patient-perceived lower-extremity function, despite the absence of superior gains in quadriceps strength.

The study observed a significant improvement in the GROC scores across all intervention groups, reflecting cumulative adaptations in pain reduction as well as improvement in strength and functional capacity. While the MI-RT group demonstrated substantial improvements by week 8 in accordance with established evidence [70], passive BFR at 70% LOP produced greater functional improvements than other groups. Given that the GROC captures patients’ perceived overall change following an intervention [42], these findings further support the superior patient-perceived recovery associated with passive BFR at 70% LOP. Conversely, the 50% LOP group reported significantly lower GROC scores, reinforcing the suggestion that higher occlusion pressures may be necessary to optimize subjective and objective outcomes in KOA rehabilitation.

Cardiovascular safety should be considered when interpreting the findings of the present study. Although participants with cardiovascular disease, uncontrolled hypertension, peripheral vascular disease, thromboembolic risk factors, and other contraindications to BFR were excluded through rigorous screening, cardiovascular responses (e.g., heart rate and blood pressure) and vascular biomarkers were not monitored during the intervention. Current evidence suggests that BFR can be safely implemented in appropriately screened individuals when individualized occlusion pressures and standardized protocols are used, with serious adverse events reported only rarely and most adverse effects being mild and transient [31, 68, 71]. Furthermore, passive BFR applied at rest has not been shown to induce clinically meaningful systemic hemodynamic alterations in healthy adults [22]. Nevertheless, these findings cannot be directly generalized to patients with KOA, particularly those with cardiovascular comorbidities. Therefore, future studies should incorporate comprehensive cardiovascular monitoring, together with vascular and coagulation biomarkers, to further establish the safety profile of passive BFR in clinical populations.

Although the findings suggest that passive BFR at 70% LOP may produce greater functional benefits than 50% LOP, they should not be interpreted as establishing a definitive dose–response relationship. Only two occlusion-pressure levels were examined; therefore, the shape, threshold, plateau, or potential nonlinearity of the pressure–response curve cannot be determined. Future studies should compare several individualized LOP levels across a broader pressure range to establish the optimal therapeutic dose and determine whether increasing pressure produces progressively greater benefits or mainly increases discomfort and cardiovascular stress.

The present study has several limitations. First, the study included adults aged 45–60 years with Kellgren–Lawrence grade II–III knee osteoarthritis, limiting the generalizability of the findings to older individuals and those with more advanced disease. Second, no follow-up assessment was performed; therefore, the long-term sustainability of the observed improvements and their influence on disease progression remain unknown. Third, cardiovascular responses (e.g., heart rate and blood pressure) were not monitored during the intervention; therefore, the hemodynamic safety of the investigated passive BFR protocols could not be directly evaluated. Fourth, the passive BFR intervention and MI-RT differed inherently in participant activity and therapist interaction. Although treatment duration and supervision were standardized across groups, differences in participant engagement and attention could not be completely controlled and may have introduced performance bias. Fifth, Kellgren–Lawrence grading was performed by a single evaluator, with confirmation from the referring orthopedic physician’s report when available; however, formal intra-rater reliability was not assessed, which may have introduced classification bias. Sixth, simple randomization resulted in a significant gender imbalance among groups. Although this imbalance was statistically adjusted by including gender as a covariate and did not alter the overall treatment effects, future trials should consider stratified randomization to improve baseline group balance. Finally, participant blinding was not feasible because of the nature of the interventions, which may have introduced response bias in self-reported outcomes.

## Conclusion

Among patients aged 45–60 years with Kellgren–Lawrence grade II–III knee osteoarthritis, both MI-RT and passive BFR at varying occlusion pressures improved quadriceps strength, pain, and functional outcomes. MI-RT produced the greatest improvements in quadriceps strength and therefore remains the preferred intervention for patients who can tolerate mechanical loading. In contrast, passive BFR at 70% LOP yielded superior improvements in lower-extremity functional ability while requiring less perceived exertion, suggesting that it may represent a feasible low-load rehabilitation alternative for patients unable to tolerate conventional resistance exercise. Passive BFR at 50% LOP also produced significant improvements; however, these were generally smaller than those achieved with MI-RT and passive BFR at 70% LOP. These findings suggest that individualized occlusion pressure may be considered when implementing passive BFR in knee osteoarthritis rehabilitation. Future studies should investigate long-term outcomes, broader age groups and disease severities, and compare different occlusion pressures with exercise-based BFR protocols to determine optimal individualized rehabilitation strategies.

### Clinical implications

The present findings indicate that, in patients capable of tolerating mechanical loading, MI-RT remains the preferred approach for optimizing quadriceps strength gains. Conversely, for individuals with pain-limited participation, passive BFR, particularly at 70% LOP, may serve as a more effective alternative for improving functional performance and patient-reported outcomes. These results underscore the importance of individualized prescription of occlusion pressure, whereby clinicians should carefully balance patient tolerance with the need to provide an adequate physiological stimulus to achieve meaningful functional improvements.

## Data Availability

Datasets used or analyzed during the current study are available upon reasonable request from the corresponding author.
